# Unmasking Villaret’s syndrome: a diagnostic challenge of glomus jugulare mimicking mastoiditis

**DOI:** 10.1097/MS9.0000000000002911

**Published:** 2025-01-21

**Authors:** Abhishek Kumar Shah, Prince Barnawal, Aayushma Kafle, Bikram Prasad Gajurel

**Affiliations:** a Maharajgunj Medical Campus; b Department of Neurology, Institute of Medicine, Tribhuvan University Teaching Hospital, Kathmandu, Nepal

**Keywords:** advanced imaging, chronic otitis media, cranial nerve palsies, glomus jugulare tumor, mastoiditis, Villaret’s syndrome

## Abstract

**Introduction:**

Villaret’s syndrome involves unilateral palsy of cranial nerves IX to XII, often with Horner’s sign, commonly caused by benign neoplasms or vascular issues. This report highlights glomus jugulare-induced Villaret syndrome, stressing careful evaluation for persistent otologic symptoms.

**Case presentation:**

A 43-year-old woman with a history of chronic suppurative otitis media (CSOM) presented with recurrent ear discharge, pain, and severe mixed hearing loss. Despite a left tympanoplasty, symptoms persisted, leading to a canal wall down mastoidectomy. Six months later, she developed facial numbness, hoarseness, and dysphagia. Physical exam showed unilateral cranial nerve palsies with Horner’s syndrome, consistent with Villaret’s syndrome. Imaging confirmed a glomus jugulare tumor. After digital subtraction angiography and balloon occlusion test, surgical resection was performed. Postoperatively, she showed gradual cranial nerve recovery and remains under follow-up.

**Discussion:**

Jugular foramen syndrome involves unilateral cranial nerve palsies, including Villaret’s syndrome, where cranial nerves IX–XII and cervical sympathetic fibers are affected. Most often linked to benign neoplasms like paragangliomas, glomus jugulare tumors are slow-growing, often benign, and predominantly affect middle-aged females. Diagnosis relies on MRI’s “salt-and-pepper” imaging, with high-resolution CT for bony involvement. Treatment, including surgery with embolization, aims to reduce cranial nerve deficits. Radiotherapy and radiosurgery are options for non-surgical candidates or recurrence cases.

**Conclusion:**

This case underscores the diagnostic challenges of Villaret’s syndrome caused by glomus jugulare. Initially masked by symptoms of chronic otitis media and mastoiditis, cranial nerve deficits eventually revealed the underlying tumor, highlighting the importance of vigilant assessment and imaging in persistent otologic cases.

## Introduction

Villaret’s syndrome is a unilateral isolated palsy of the last four cranial nerves, associated with a Horner’s sign^[1]^.The most common causes of Villaret syndrome are benign neoplasms or vascular abnormalities with less common causes being secondary to metastases arising from lung and prostate carcinoma^[1]^. Glomus jugulare is a rare, slow-growing neuroendocrine paraganglioma of the head and neck that arises within the jugular foramen and is localized to the jugular fossa in the temporal bone of the skull base^[2,3]^.

This case is unique in that the glomus jugulare tumor, initially overlooked due to its size, later manifested with classic features of Villaret syndrome, emphasizing the importance of careful assessment in patients with persistent otologic symptoms. This case report is written as per SCARE guidelines^[4]^.

## Case description

**Figure 1.**
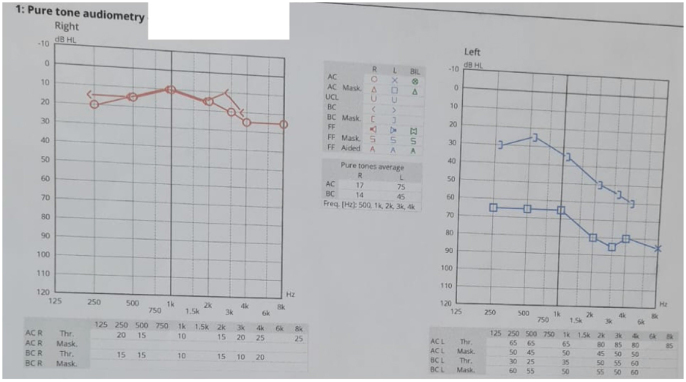
Pure tone audiometry showing normal hearing sensitivity in the right ear, with air conduction (AC) and bone conduction (BC) thresholds within normal limits. The left ear demonstrates severe mixed hearing loss with air conduction thresholds around 65–85 dB and bone conduction thresholds around 45–55 dB, indicating mixed hearing impairment.

A 43-year-old woman presented with a three-month history of recurrent ear discharge and pain and was diagnosed with left-sided CSOM. She initially underwent a left tympanoplasty to treat the condition, but her symptoms persisted postoperatively. Six months later, she returned with similar complaints. Audiometric testing revealed severe mixed hearing loss in the left ear (Fig. 1), and a CT scan of the temporal bones showed soft tissue densities in the middle ear cavity and mastoid air cells, consistent with mastoiditis. As a result, she underwent a left canal wall down mastoidectomy.

Six months after the mastoidectomy, she was admitted to the emergency department with a three-month history of facial numbness and pain, along with 2-month history of dysphagia, hoarseness, and slurred speech.

**Figure 2.**
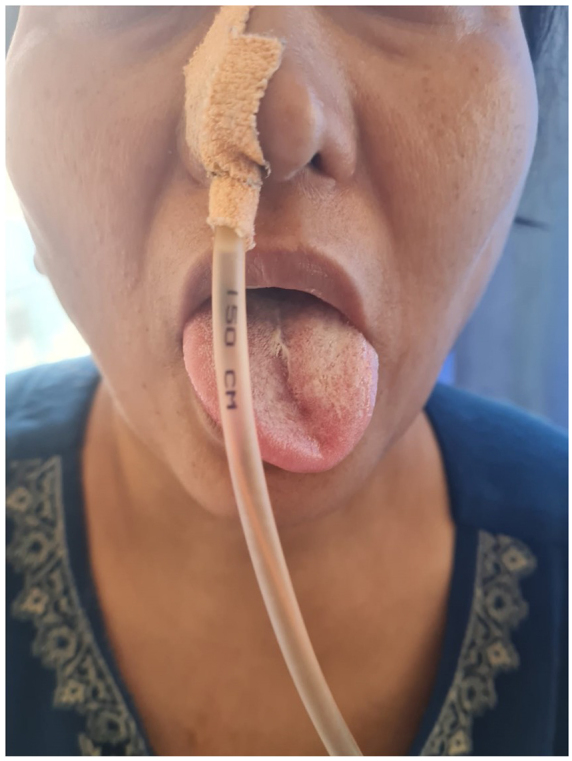
Tongue deviation to the left on protrusion, indicative of hypoglossal nerve palsy. A nasogastric (NG) tube is in place for feeding due to dysphagia, resulting from cranial nerve IX (glossopharyngeal) and X (vagus) palsy.

**Figure 3.**
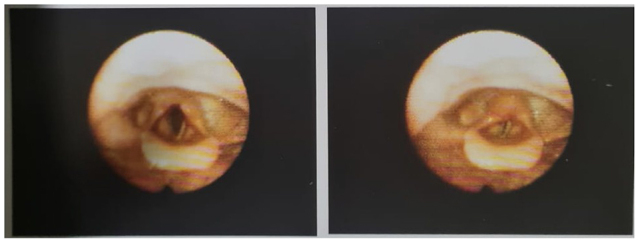
Flexible endoscopy images showing left vocal cord immobility, consistent with a diagnosis of left vocal cord paralysis.

On physical examination, there was evidence of cranial nerve VII and VIII involvement, with the angle of the mouth deviated to the right and left-sided facial palsy (Grade II). The tongue deviated to the left on protrusion (Fig. 2), suggesting CN XII involvement alongside uvular deviation to the right, indicating vagus nerve (CN X) impairment. Neck examination revealed weakness of the left sternocleidomastoid muscle, suggesting accessory nerve (CN XI) involvement. Direct laryngoscopy revealed a left vocal cord paralysis (Fig. 3). Anisocoria and Horner syndrome on the left side were present. Thus, all these signs evoked a unilateral IX-XII cranial nerve paralysis, with a Horner’s syndrome. This association of symptoms is known as Villaret’s syndrome.

**Figure 4.**
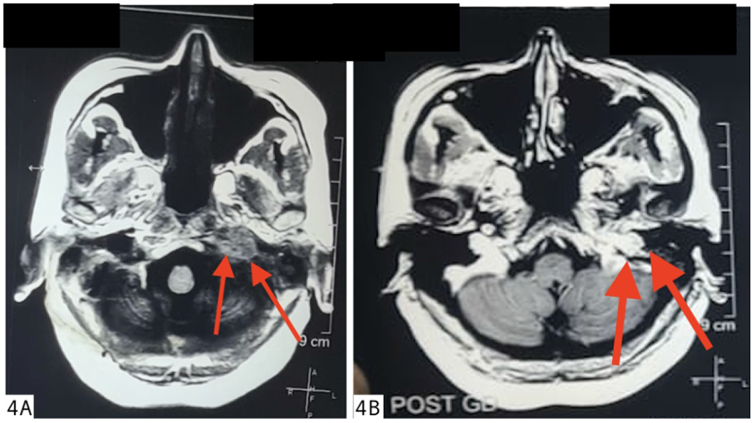
**(A) and (B**): Axial T1-weighted (left) and T1-weighted post-gadolinium (right) MRI images of the skull base. The red arrows indicate a soft tissue lesion on the left side, which appears low signal on T1W (left) and shows heterogeneous, avid enhancement post-gadolinium (right). The lesion causes lytic destruction of the left mastoid air cells and extends into the middle ear cavity.

**Figure 5.**
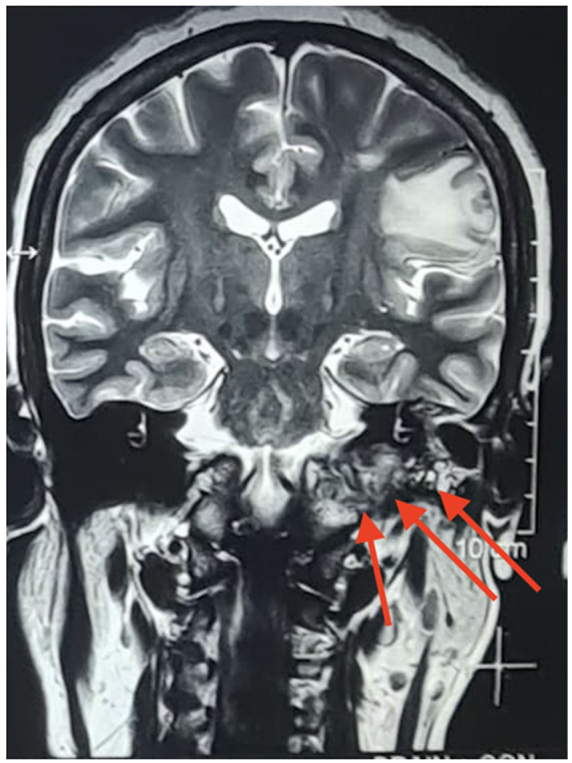
Coronal T2-weighted MRI image showing a soft tissue lesion at the base of the skull on the left side (indicated by red arrows). The lesion appears iso-intense on T2W and causes lytic destruction of the left mastoid air cells, with extension into the middle ear cavity.

**Figure 6.**
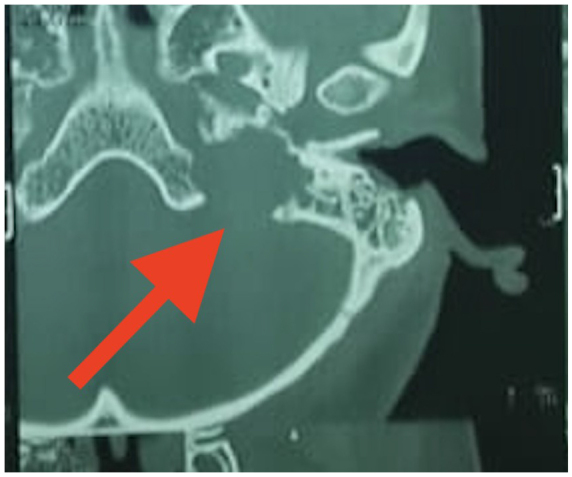
HRCT of the temporal bone showing erosion of the left jugular fossa and sinus plate with a mass extending into the middle ear cavity and carotid canal involvement (indicated by red arrows).

A cerebrospinal fluid cytology report revealed no abnormalities. TB tests and other infections were ruled out. T1-weighted (T1W), T2-weighted (T2W), and T1-weighted gadolinium-enhanced MRI sequences were done. There was a soft tissue lesion in the base of the skull on the Lt. side. Lesion is low signal on T1W iso-signal on T2W and demonstrates heterogeneous but avid post gadolinium enhancement (Fig. 4A and 4B). The lesion caused lytic destruction of the left mastoid air cells, extended into the middle ear cavity (Figs. 4A, B and 5).HRCT of the temporal bone showing erosion of the left jugular fossa and sinus plate with a mass extending into the middle ear cavity and carotid canal involvement (Fig. 6).These findings were consistent with Class C glomus jugulare tumor.

She was initially evaluated using digital subtraction angiography (DSA) to assess the vascular nature of the lesion and balloon occlusion test (BOT) was performed to evaluate the adequacy of collateral blood supply to the left jugular vein. The test revealed sufficient collateral circulation from the right side. Based on the DSA and BOT, it was determined that surgical resection of the tumor was feasible. The patient underwent surgery via an infratemporal fossa type A approach.

The patient’s postoperative course was uneventful, with gradual recovery of lower cranial nerve function over the following weeks. Follow up imaging after post operation confirmed the absence of residual tumor, and the patient is currently under routine surveillance for tumor recurrence.

## Discussion

Jugular foramen (Vernet) syndrome typically presents with hoarseness and dysphagia that may be associated with unilateral pharyngeal pain. Cranial nerves IX through XI are typically affected. Thereby, patients exhibit paralysis of the ipsilateral palate, vocal fold, sternocleidomastoid, and upper trapezius^[5]^. There are several variations of jugular foramen syndrome related to the location of the skull base mass including Jackson syndrome (dysfunction of cranial nerves X, XI, and XII), Tapas syndrome (dysfunction of cranial nerve X and XII), Collet–Sicard syndrome (dysfunction of cranial nerves IX, X, XI, and XII), and Villaret syndrome (dysfunction of cranial nerves IX, X, XI, and XII, as well as the cervical chain sympathetics)^[6]^. The most common causes of Villaret syndrome are benign neoplasms, such as meningioma, paraganglioma, and schwannoma, or vascular abnormalities, such as internal carotid aneurysm. Less commonly, metastasis has been reported as a cause^[1]^.

Glomus jugulare tumors arise from the paraganglia cells located in the adventitia wall of the jugular bulb, within the jugular foramen. They have slow growth and are usually benign. Only 1% to 5% are malignant^[2]^. Eighty percent of paragangliomas are sporadic, with the rest exhibiting a hereditary component^[7]^. Inherited forms will present with an earlier onset of symptoms and can be bilateral; however, familial forms have a decreased pattern of malignancy when compared to sporadic counterparts^[7]^.

Early-stage paragangliomas present with signs and symptoms related to their location. Specifically, most of these will be related to the involvement of the middle-ear cleft. These neoplasms tend to spread through the hypotympanic air cell tract, around the jugular bulb, inferior petrosal sinus and carotid artery into the jugular foramen and posterior fossa^[7]^. Further extension of the tumor through the facial recess, can result in facial nerve encasement and paralysis^[7]^.

Glomus jugulare PGLs usually diagnosed during the fourth to fifth decades of life with a female predominance (female to male ratio = 5:1)^[8]^. Patients present most commonly with hearing loss, pulsatile tinnitus, and lower cranial nerve neuropathy^[9,10]^,the most common being pulsatile tinnitus, followed by hearing decline^[8]^. Lower cranial nerve deficits are encountered in up to 30% of cases and can present with neurological deficits, resulting in facial palsy, dysphagia, hoarseness, shoulder weakness, and tongue deviation and are usually seen with larger tumors that extend through the medial wall of the jugular bulb^[9]^. Other symptoms include earache, ear discharge, and ear bleeding.

Audiologic testing pure tone and speech audiometry in glomus jugulare patient mostly reveals mixed hearing loss, though the extent of sensorineural impairment is variable^[11]^. In our case as well, she had severe mixed hearing loss with air conduction thresholds around 65–85 dB and bone conduction thresholds around 45–55 dB, indicating mixed hearing impairment.

The MRI reveals a lesion with a low signal intensity on T1-weighted imaging and high signal intensity on T2-weighted imaging^[12]^. Post-contrast T1-weighted imaging with gadolinium (T1 C + Gd) shows marked and intense enhancement^[12,13]^. A characteristic “salt and pepper” appearance is noted on both T1 and T2 sequences, where “salt” represents blood products from hemorrhage or slow flow, and “pepper” indicates flow voids caused by the lesion’s high vascularity. However, this appearance is occasionally observed in other hypervascular lesions, such as hypervascular metastases, and is typically absent in smaller paragangliomas. Our differential diagnosis also included jugular schwannoma. Unlike glomus jugulare, it has sharply demarcated smooth bony margins, poor vascularity on angiography and no salt and pepper appearance^[14]^. Our case had classical MRI imaging findings of glomus jugulare similar to what is given in previous literature^[15]^. On high-resolution computed tomography (HRCT) of the temporal bone^[15]^, glomus jugulare causes an irregular erosion of the floor of the hypotympanum with a characteristic “moth-eaten” appearance. In our case as well, there was erosion of the left jugular fossa and sinus plate with a mass extending into the middle ear cavity and carotid canal involvement. Our findings also support previous literature that recommends MRI as the gold standard for detecting soft tissue and vascular characteristics, while high-resolution CT is essential for assessing bone involvement in paragangliomas^[15]^.

Intra-arterial DSA has been proved to be very satisfactory for mapping of tumor blood supply, simplifying preoperative embolization. It also assesses the feasibility of a safe and effective resection^[16]^. Angiography also helps differentiate paragangliomas from other pathologies and demonstrate the tumor blush and the feeding vessels, which can then be embolized. Balloon test occlusion studies helps to decide whether occlusion of the vein would be tolerated, thus helping to reduce the risk of ischemic complications^[16]^. Our patient was initially evaluated using DSA and BOT. The test revealed sufficient collateral circulation from the right side. Based on the DSA and BOT, it was determined that surgical resection of the tumor was feasible.

## Treatment

The management of glomus tumors continues to be a challenge. There are still many controversies regarding optimal treatment. Tumor classifications described by Fisch and Glasscock are the most commonly used^[16,17]^. The treatment of this tumor involve surgical resection, conventional radiation therapy, stereotactic radiosurgery (SRS), therapeutic embolization, or a combination of these modalities^[10]^. For young, healthy patients with functional cranial nerve deficits, the mainstay treatment of choice is surgical removal. Preoperative embolization is generally performed 24–72 hours before surgery^[16]^. In about 80% of patients, complete resection may be accomplished, but it may result in debilitating cranial neuropathy. Subtotal resection has been used more frequently to minimize morbidity and improve symptoms associated with the disease^[18]^. Both gross and subtotal surgical resection carry a significant risk of morbidity, and as a result, SRS has become an increasingly popular treatment^[3]^. A systematic review of the literature shows that EBRT and SRS are comparable to surgical intervention in patients with jugular paragangliomas^[19]^.

Modalities for radiotherapy include standard fractionated radiotherapy and SRS. It can be offered as an adjunctive to limited surgery or as the primary treatment modality in poor surgical candidates or patients with bilateral disease providing up to 90% control rate^[7,20]^. However, radiotherapy is contraindicated in the presence of intracranial invasion or extensive osteomyelitis^[19,21]^. In the elderly and medically unfit subjects with minimal symptoms, a close observation is an alternate option, especially in patients without brainstem compression or concerns for malignancy^[22,23]^.

Endovascular embolization, as a single modality of treatment, is considered palliative^[7]^. Complete obliteration of glomus jugulare tumors with the use of embolization is very difficult and is prone to revascularization and is not beneficial in terms of alleviating clinical symptoms^[24]^. Preoperative embolization of the tumor can lead to a decrease in the duration of surgery, as well as a reduction of operative estimated blood loss^[7]^. Therefore, the treatment decision should be individualized in each patient. Since our patient was young, healthy with functional cranial nerve deficit, she was managed using preoperative embolization followed by surgical resection through infratemporal fossa type A approach. It successfully resected the tumor, with gradual recovery of cranial nerve function postoperatively without any complication after 6 months of follow-up.

## Conclusion

To our knowledge, there is no similar report to date of a glomus jugular paraganglioma with presentation as Villaret’s syndrome, and mastoiditis. This case highlights the diagnostic complexities of Villaret’s syndrome secondary to a glomus jugulare tumor, a rare and often overlooked cause of unilateral lower cranial nerve palsies. We identified a patient with recurrent ear discharge and ear pain, who was initially diagnosed with mastoiditis. Initial symptoms of chronic otitis media and mastoiditis masked the underlying tumor, leading to a delay in diagnosis until the characteristic presentation of multiple cranial nerve deficits emerged. Later, imaging studies, particularly MRI and HRCT, were instrumental in revealing the lesion’s extent, location, and vascular involvement, ultimately guiding the diagnosis. A high index of clinical suspicion is important if patients remain symptomatic after the treatment of mastoiditis. Here, we emphasizes the need for thorough assessment and advanced imaging in patients with persistent otologic symptoms and progressive cranial nerve involvement, underscoring the significance of early and accurate diagnosis in managing glomus jugulare tumors presenting with Villaret’s syndrome.

## Data Availability

All available data are within manuscript itself.
